# Developing the *Understanding Palliative Care* Module: A Quality Improvement Initiative Incorporating Public, Patient, and Family Caregiver Perspectives

**DOI:** 10.3390/curroncol32040221

**Published:** 2025-04-10

**Authors:** Patricia Biondo, Mary-Ann Shantz, Yuanjie (Bill) Zheng, Miranda Manning, Louise Kashuba

**Affiliations:** 1 Division of Palliative Medicine, University of Calgary, Calgary, AB T2N 5G2, Canada; pbiondo@ucalgary.ca; 2 Covenant Health Palliative Institute, Edmonton, AB T5K 2K4, Canada; 3 Volunteer Public Advisor, Covenant Health Palliative Institute, Edmonton, AB T5K 2K4, Canada

**Keywords:** palliative care, public awareness, education, caregivers

## Abstract

Improving public awareness of palliative care is crucial for improving access to, and uptake of, palliative care, which has demonstrated benefits for patients and health systems. However, there is a lack of engaging, accessible educational palliative care resources designed for public audiences. As part of a larger quality improvement initiative to strengthen awareness of palliative care, we developed “*Understanding Palliative Care*”—an innovative, online educational module incorporating best practices for defining and promoting palliative care to a public audience. An expert working group with representation from nursing, medicine, social work, instructional design, and care navigation advised on the development of the module. Incorporating the perspectives of Albertans with lived palliative care experience was deemed essential by the working group. We identified three Albertans (one patient and two family caregivers) of diverse ages and cultural backgrounds who had personally benefitted from palliative care and consented to record virtual interviews. We incorporated multiple interview segments into the module that highlight the physical, emotional, social, and spiritual support provided by palliative care. Finally, a panel of thirteen public volunteers provided feedback on the content, design, and navigation of the draft module. The *Understanding Palliative Care* module fills an important gap in Alberta, providing a free, online, evidence-based, and engaging educational tool to improve public awareness and understanding of palliative care.

## 1. Introduction

Palliative care focuses on improving the quality of life for people living with serious illnesses. Despite extensive research evidence supporting the benefits of palliative care (e.g., improved symptom management, quality of life, advance care planning, care coordination, and even survival) [1,2,3,4], many people who could benefit from palliative care receive it too late or not at all [5,6]. This is often due to the widely-held misconception that palliative care is only for people at the very end of life [1,7,8]; clinicians are often reluctant to raise the topic for fear of upsetting patients [9,10], and patients may refuse palliative care because they do not see it as applicable to their situation or understand how it can help [11,12].

Improving public awareness of palliative care has been identified as a necessary component of improving access to, and uptake of, palliative care [8,13,14]. According to an Ipsos Canada survey completed in 2016, only 58% of Canadians have a basic understanding of palliative care [15]. Recent policy documents from many countries, including Canada, call for public awareness campaigns and education about palliative care [16,17,18,19,20]. In 2020, the Covenant Health Palliative Institute embarked on a multi-year, multi-sector palliative care public awareness initiative in Alberta, Canada [21]. The initiative aims to improve public awareness and understanding of palliative care through tools and resources developed and implemented in collaboration with community partners. Our search for an engaging, accessible, evidence-based educational resource on palliative care, designed specifically for a public audience, identified this as an important gap—in Canada and internationally. The purpose of this work was thus to develop such a tool for use in Alberta, Canada.

## 2. Materials and Methods

### 2.1. Literature Review and Environmental Scan

A literature review and environmental scan were conducted to identify existing tools or resources that could be adapted to the Alberta context. A literature search on Compassionate Communities and/or public health approaches to palliative care was conducted using MEDLINE, PubMed, CINAHL, Cochrane Library, PsycINFO, Portal of Geriatric Online Education, and Google Scholar databases. In addition, systematic searches of the grey literature and palliative care websites were conducted to identify tools and resources related to palliative care and advance care planning. We sought to find evidence-based resource(s) designed for a public audience that were free or low-cost, promoted active user engagement, and were publicly accessible. Conversely, we excluded resources whose primary audience was clinicians, patients and families, caregivers or volunteers that were developed for use in a clinical setting, that came at a high cost to the end user, or that provided information in a passive format (such as websites or informational booklets/brochures).

An online educational module developed by the All Ireland Institute of Hospice and Palliative Care, called “Introduction to Palliative Care”, was identified as a potential candidate, as it was designed for a public audience, featured interactive content, and incorporated video interviews of clinicians and caregivers with palliative care experience [22]. Permission was received from the All Ireland Institute of Hospice and Palliative Care to adapt their resources (personal communication with Karen Charnley, Director, All Ireland Institute of Hospice and Palliative Care, December 2021).

### 2.2. Development of the Alberta Module

#### 2.2.1. Process

We convened an expert working group to advise us on adapting the Irish module for use in Alberta. The working group consisted of palliative care clinicians (one physician, two clinical nurse specialists in palliative care, one palliative resource nurse, one nurse practitioner, and one social worker), one staff member of a community hospice palliative care society, a Learning and Development Consultant with Covenant Health, and one public advisor. The working group was facilitated by two Covenant Health Palliative Institute project staff.

**Figure 1:**
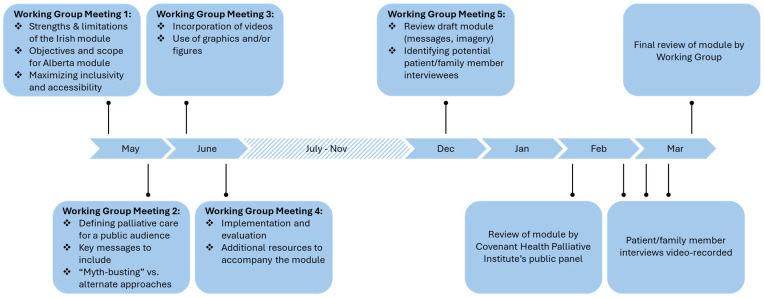
Process of developing the *Understanding Palliative Care* module: timeline and working group discussion topics.

Five working group meetings were held between May and December of 2022 to discuss the development of the Alberta module (see Figure 1 for timeline and meeting agendas). Evidence-based resources on messaging palliative care for public audiences (e.g., the Center to Advance Palliative Care’s MOTIVATE: Marketing and Messaging Palliative Care Toolkit [23], the Serious Illness Messaging Toolkit [24], and academic articles [25,26]) were shared with the group to inform the discussion. In February 2023, a draft module was presented for review by the Covenant Health Palliative Institute’s public panel, a group of 13 public volunteers who were convened to advise on the Institute’s public awareness initiatives. A recording of this meeting was made available to panel members who were unable to attend. Panel members were invited to provide feedback on the draft text, graphics, navigation, and evaluation survey (video content was not ready/presented for review). Feedback was gathered during the meeting as well as through a post-meeting survey and used to improve the module. In March 2023, the near-final module was shared with the working group and feedback was solicited via email or an online survey.

#### 2.2.2. Design

**Figure 2:**
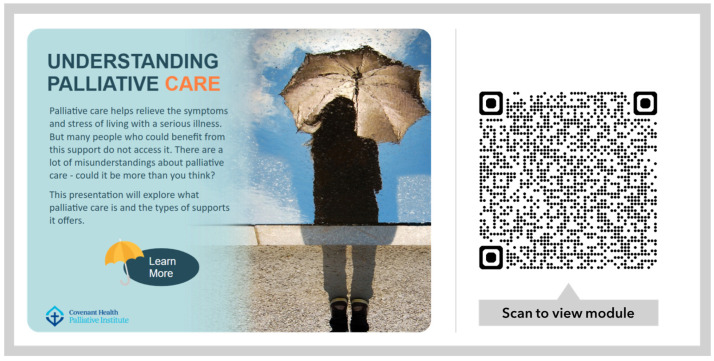
The *Understanding Palliative Care* module.

The module was developed using Storyline 360 software by Articulate. A storyboard containing draft text, sample images, and notes on branching and interaction features was created for review by the project team. After feedback was received on the storyboard, a draft of the module was uploaded to our organization’s learning management system to allow for review in a test environment. The final module was published on the web and made available on the Covenant Health Palliative Institute’s website for public access (Figure 2).

#### 2.2.3. Video Content

The Irish module incorporated several short video clips of interviews with clinicians and caregivers with palliative care experience. However, because these videos were obviously specific to the Irish context, we deemed them unsuitable for inclusion in the Alberta module. The working group reviewed a number of existing videos (created locally, nationally, and internationally) in the event that existing content could be leveraged and we could avoid duplicating work if appropriate videos already existed. However, we were unable to find enough video content with a consistent look and feel, which aligned with our key messages, reflected sufficient diversity (e.g., of subjects, illnesses, and settings), and was not obviously specific to a geographic area outside of Alberta. To that end, we decided to create our own videos.

We were able to identify three Albertans (one patient and two family caregivers) of diverse ages (young adult, middle-aged, and senior), cultural backgrounds (Chinese and European), and illnesses (cancer and non-cancer), who had personally benefitted from palliative care and consented to record virtual interviews. Two interviewees were identified through the Institute’s public panel, and one interviewee was approached by one of our working group members. An interview guide was developed by project staff (Table 1), with questions designed to explore the physical, emotional, social, and spiritual support provided by palliative care. Questions were shared with the interviewees in advance to help them prepare, but they were advised not to script their responses. Interviews were conducted by project staff (MS) and recorded via Zoom in January and February of 2023. Interviewees were given the option to appear in the videos or to participate anonymously; one interviewee chose the latter, and her video segments incorporated stock photography and video footage. All interviewees signed a consent form for the collection and disclosure of their interviews.

Video footage was reviewed by two authors (MS and MM). A number of short clips from each interview, highlighting the benefits and support received from palliative care, were chosen for incorporation into the module.

### 2.3. Evaluation

An evaluation survey for module users was created and managed using REDCap electronic data capture tools [27] hosted by Alberta Health Services. The survey link is accessed directly from the last screen of the module. The primary objective of the survey is to assess the effects of the module on users’ understanding of and attitudes toward palliative care. Secondary objectives are to learn where people heard about the module, who we are reaching with the module, and suggestions for improvement.

## 3. Results

### 3.1. Working Group Feedback

The key recommendations provided by working group members were as follows: (1) use plain language; (2) move away from the “myth-busting” approach of the Irish module and focus instead on positive key messages; (3) feature Albertans with lived experience of palliative care; and (4) use friendly, engaging graphics and navigation. To effectively respond to working group feedback, we decided to develop the Alberta module from the ground up rather than closely replicate the Irish template, although it continued to serve as inspiration.

#### 3.1.1. Use Plain Language

The working group strongly recommended using plain language at a literacy level intelligible to most of the general public (~grade 6–8 level). They encouraged avoiding the use of medical jargon and advised against using the World Health Organization’s definition of palliative care, despite its widespread use in the clinical setting. Instead, they recommended using a shorter, simpler definition that avoided the phrase “life-limiting”, which members felt was not easily understood by the public. We were also advised to not equate palliative care with end-of-life care nor use these terms interchangeably, as this would undermine some of the key messages (i.e., that palliative care can benefit people starting at the time of diagnosis with a serious illness, and that it can be received along with other treatments). Following this advice, the module opens with the explanation that palliative care “helps relieve the symptoms and stress of living with a serious illness”.

#### 3.1.2. Focus on Positive Key Messages

**Figure 3:**
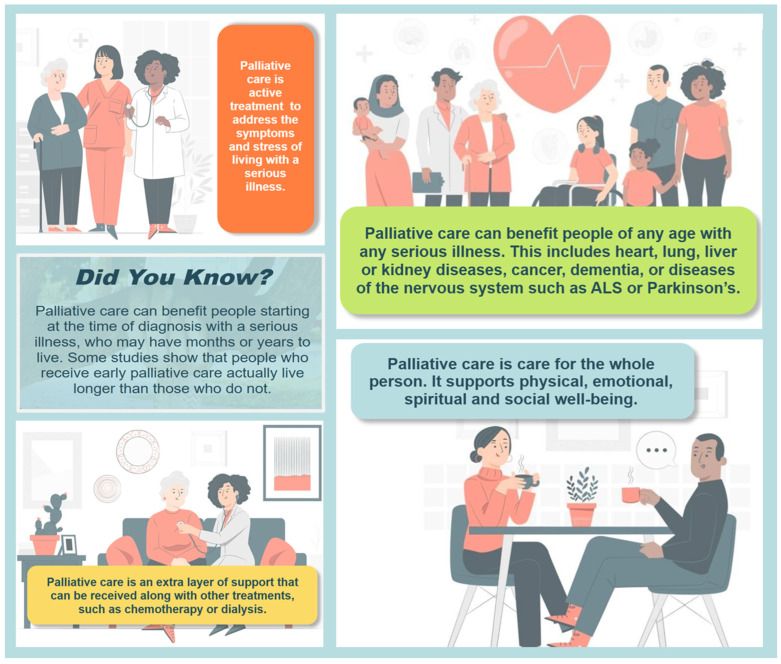
Key messages highlighted in the module.

The working group encouraged focusing on positive key messages about palliative care rather than using a “myth-busting” approach, which can have a negative tone and may inadvertently perpetuate common misunderstandings. The group also suggested highlighting a small number of key messages and weaving these throughout the module, rather than offering a detailed definition of palliative care at the beginning and potentially overwhelming the user with too much information. The final key messages incorporated into the module are shown in Figure 3.

#### 3.1.3. Feature Albertans with Lived Experience of Palliative Care

The working group deemed it essential that we incorporate personal stories from local people with lived palliative care experience who could speak to the benefits they received from palliative care, both to help users connect emotionally with the topic and to provide concrete examples of how palliative care helped. Conversely, the working group did not feel it was necessary to incorporate interviews with experts or clinicians. Short video clips were recommended to support engagement, to vary the format in which information was presented, and to facilitate the story-telling aspect. Videos were also seen as an additional opportunity to convey diversity (e.g., of ages, ethnicities, illnesses, and settings).

#### 3.1.4. Use Friendly, Engaging Graphics and Navigation

Building the module from the ground up provided an opportunity to incorporate engaging graphics and navigation, as suggested by the working group. The Alberta module incorporates bright colours and a mix of cartoon imagery and photos that highlight a diversity of people and settings. The working group advised us to avoid images of medical settings to align with recent research supporting the use of aspirational images that show people living well with a serious illness [24]. We also drew inspiration from an article shared by a working group member, Zimmermann and Mathews’ “Palliative Care Is the Umbrella, Not the Rain-A Metaphor to Guide Conversations in Advanced Cancer” [26], and incorporated a colourful umbrella theme throughout the module as a metaphor to convey that palliative care can provide shelter from the symptoms and stress of living with a serious illness.

### 3.2. The Understanding Palliative Care Module

The *Understanding Palliative Care* module is open access and freely accessible on the Covenant Health Palliative Institute’s website [28]. Key features of the module include the following: a simple, plain language definition of palliative care that is expanded upon throughout the module; a focus on five positive key messages; an emphasis on the holistic supports provided by palliative care; video interview segments featuring three Albertans who personally benefitted from palliative care; bright colours and images; and an emphasis on diversity.

The module is interactive and self-directed: users click on different icons to progress through the module at their own speed. The centrepiece of the module highlights the holistic support provided by palliative care, organized according to physical, emotional, social, and spiritual care domains. Each of these domains describes challenges that may be faced by someone living with a serious illness, how palliative care can help with these challenges, and 1–2 video clips that exemplify the support palliative care provided in this area from the perspective of someone with lived experience.

The module takes approximately 20 min to complete, including seven short videos comprising a total of 10 min of content, and one video compilation near the end in which the subjects reflect on “what do you think everyone should know about palliative care?”. A one-page, downloadable handout was developed to accompany the module, which summarizes the module’s key messages and provides web links to additional resources.

### 3.3. Dissemination of the Module

The *Understanding Palliative Care* module was launched via a social media campaign, e-blast, and online articles in May 2023 to coincide with Canada’s National Hospice Palliative Care Week. We also reached out to individual organizations within key stakeholder groups (e.g., hospice palliative care societies, disease-specific support organizations, senior-serving organizations, faith and cultural groups, and libraries), as well as our provincial healthcare system, with a targeted request to share the module with their members. This could include linking to the module from their website, including information about the module in their communications (e.g., newsletters and brochures), or inviting us to present to their membership.

### 3.4. Evaluation

Due to the format by which the *Understanding Palliative Care* module is embedded on the web, we are unable to obtain web analytics on how many people have accessed the module. While this limits our evaluation, we wanted to ensure easy access to the module with no login or permissions required. As a proxy measure for the number of module users, we periodically capture the number of visitors to the webpage that houses the *Understanding Palliative Care* module. To date, our web analytics indicate we have had more than 3000 visits to this webpage.

Uptake of the evaluation survey has been limited, but the data suggest the module is improving knowledge of and attitudes toward palliative care: 92% of survey respondents (22/24) indicated that the module improved their understanding of palliative care, and 100% of survey respondents (24/24) would consider palliative care for themselves or someone close to them. The free text responses have been overwhelmingly positive, e.g., “I appreciated the interactive engaging style of this module and the combination of professional information and personal stories”; “Well done—love the visuals, stories, and interactive nature of this module!”; “Beautiful presentation. I liked how interactive it was; visually pleasing with nice colours and gentle tones”.

## 4. Discussion

The *Understanding Palliative Care* module fills an important gap, providing a free, online, evidence-based, and engaging public-facing tool to improve public awareness and understanding of palliative care. The module incorporates best practices in defining and promoting palliative care to a public audience, which include the following: using simple, plain language; focusing on the benefits; incorporating stories from real people who have benefitted from palliative care; and using imagery that is aspirational for the intended audience and shows people living well with serious illnesses [23,24,29]. Additionally, we followed the recommendations of the MOTIVATE toolkit to “not define palliative care by what it is not” (i.e., “Palliative care is not the same as end-of-life care”) as people tend to remember words in proximity and so may remember the negative you are trying to distinguish from [23].

Traditionally, medical professionals have led public education on healthcare topics due to their recognized expertise [30]. However, individuals with lived experiences provide unique insights that have been shown to enhance educational outcomes [30]. Despite these benefits, educational resources on palliative care rarely integrate the perspectives of those who have directly accessed these services, perpetuating a gap in representation.

The *Understanding Palliative Care* module sought to address this gap by collaborating with local Albertans who had personal experience with palliative care, either for themselves or family members. These participants engaged in a co-design process, contributing to the development of questions, video content, and the final structure of the module. This collaborative approach aligns with existing research demonstrating that the inclusion of people with lived experiences fosters trust and engagement in educational materials [30].

We sought to include Albertans from diverse backgrounds to ensure representation, and to address another common limitation in palliative care education. For instance, one co-researcher, Y.Z., shared his experience at the age of 18 accessing palliative care for his mother, who was living with a non-cancer illness. Including voices from underrepresented groups not only addresses a gap in representation but also helps to build trust and rapport with viewers by presenting relatable, real-world experiences.

The module’s emphasis on positive messaging about palliative care and focus on quality of life throughout a serious illness implicitly distinguishes palliative care from hospice or end-of-life care [31]. Feedback from module users suggests that lived experiences shared by patients and families help address misconceptions while building credibility, reinforcing the importance of including these in educational content. These findings suggest that incorporating diverse, patient-centred perspectives can enhance public understanding of palliative care as a holistic and supportive service.

Future considerations for the *Understanding Palliative Care* module include modifications to improve accessibility, as well as a more fulsome evaluation. Accessibility can be improved by adding a voiceover component; this will also allow for closed captioning and language translation. Future research could formally test the efficacy of the module to improve palliative care understanding and acceptability among module users.

## 5. Conclusions

The *Understanding Palliative Care* module is an engaging, accessible, evidence-based educational resource on palliative care that is designed specifically for a public audience and features individuals who have personally benefitted from palliative care. With a focus on positive messaging and the benefits of palliative care, the module may help to alleviate fear or hesitancy associated with palliative care. Others developing public education may find inspiration in our process of reviewing the literature, forming an expert working group to advise us, interviewing people with lived experience, and seeking feedback from members of the public. The *Understanding Palliative Care* module can be used with the public, patients, and family caregivers to improve public awareness and understanding of palliative care to ultimately improve access to and uptake of palliative care.

## Figures and Tables

**Table 1:** Interview guide for patient/family caregiver interviews.

Interview Guide
How has palliative care helped/benefitted you/those close to you? What physical symptoms were/are challenging for you/your family member, and how has palliative care helped address those symptoms? Are you able to share some of the emotions you have experienced during your illness/your family member’s illness? Did palliative care help you cope emotionally with your/your family member’s illness? If so, in what way? At the time of your/your family member’s diagnosis, or in the time that followed, did you/they have spiritual questions or concerns? If yes, have you/they received support to help you/them on your/their journey? Did palliative care play a role? How did your/your family member’s illness impact you socially? Has palliative care provided support in this area? Has palliative care provided support to your family or caregivers? How has it helped them? What is something you have learned about palliative care since you/your family member received it? What do you think everyone should know about palliative care?

## Data Availability

No new data were created or analyzed in this study. Data sharing is not applicable to this article.
